# Immobilization of alginate C-5 epimerases using *Bacillus subtilis* spore display

**DOI:** 10.1128/aem.00298-25

**Published:** 2025-04-03

**Authors:** Jan Benedict Spannenkrebs, Agnes Beenfeldt Petersen, Finn Lillelund Aachmann, Johannes Kabisch

**Affiliations:** 1 Department of Biotechnology and Food Science, NTNU Norwegian University of Science and Technology 205785, Trondheim, Norway; Georgia Institute of Technology, Atlanta, Georgia, USA

**Keywords:** alginate, epimerase, *Bacillus subtilis*, spore display, nuclear magnetic resonance

## Abstract

Alginates are the most abundant polysaccharides found in brown seaweed, composed of (1→4)-linked β-D-mannuronate (M) and its C-5 epimer, α-L-guluronate (G). The G-blocks of alginate possess viscosifying and gelling properties, making alginates valuable industrial polysaccharides. Alginate epimerases are enzymes epimerizing M to G, enhancing the usability and value of alginate. The three alginate epimerases AlgE1, AlgE4, and AlgE6 were immobilized using *Bacillus subtilis* spores displaying the epimerases fused to the spore crust protein CotY. To our knowledge, this is the first display of immobilized alginate-modifying enzymes. Activity assays of the four AlgE4-displaying spore strains showed that AlgE4 produced MG-blocks from polyM alginate. AlgE4 was tested linked by its N- and C-termini. Two linkers with different flexibility were tested, both containing a TEV protease cleavage site. Immobilizing alginate epimerases on *B. subtilis* spores resulted in a recyclable system that is easy to isolate and reuse, thus opening possibilities for industrial application. Recyclability was demonstrated by performing five consecutive reactions with the same batch of AlgE4 spores, with the spores retaining 24% of the starting activity after four rounds of reuse. TEV cleavage of spore-displayed enzyme was optimized using spores displaying a green fluorescent protein, and these optimized conditions were used to cleave AlgE4 off the spores. The cleavage of four AlgE4-displaying spores was successful, but cleavage efficiency varied depending on which terminus of AlgE4 was fused to CotY.

**IMPORTANCE:**

Seaweed is a scalable resource that requires no fresh water, fertilizer, or arable land, making it an important biomass for bioeconomies. Alginates are a major component of brown seaweed and are widely used in food, feed, technical, and pharmacological industries. To tailor the functional properties of alginates, alginate epimerases have shown to be promising for postharvest valorization of alginate. This study investigates an efficient and easy method to produce immobilized alginate epimerases, thus opening new industrial use cases. In this study, the alginate epimerases are immobilized on the surface of *Bacillus subtilis* spores. The bacterium forms spores in reaction to nutrient starvation, which are highly resistant to external influences and can be repurposed as a stable protein display platform for numerous applications due to its ease of genomic manipulation and cultivation.

## INTRODUCTION

Alginates are the most abundant polysaccharides in brown algae and are synthesized by some bacterial species of the *Azotobacter* and *Pseudomonas* genus (1–4). Alginates are commercially harvested from brown seaweed and have a wide range of industrial applications, including pharmaceutical and technical uses, as well as additives in the feed and food industries (5, 6). Worldwide, the market for alginates is approximately 35,000 tonnes annually and growing, but suitable seaweeds for alginate production have become more difficult to obtain (7). The usability and value of alginates depend on their composition and chain length (6), both of which can be tailored using alginate-modifying enzymes.

Alginates are a family of linear, anionic polysaccharides comprised of (1→4)-linked β-D-mannuronate (M) and its C-5 epimer α-L-guluronate (G) (8). Alginate chains can thus be divided into blocks of M (polyM), blocks of G (polyG), and alternating MG-blocks (polyMG) (9). G-blocks of alginate can bind divalent metal ions (e.g., Ca^2+^, Sr^2+^) described by the so-called egg-box model (10), forming alginate hydrogels. The gelling ability of alginates along with their ability to bind water and increase viscosity has resulted in alginate being widely used in industry, e.g., as a food thickening agent or for pharmaceutical applications (6, 11, 12). The M-residues do not have the same chelating ability, so high G-content alginates are the most commercially valuable (12). Alginates are polymerized as polyM and then epimerized post-polymerization to introduce G-residues by mannuronate C-5 epimerases (alginate epimerases) (13, 14).

Bacterial alginate epimerases are divided into two types: AlgG-type and AlgE-type (15). The AlgGs are periplasmic epimerases that introduce single G-residues in polyM during translocation of the polysaccharide (16). AlgEs are Ca^2+^-dependent extracellular enzymes (4, 17). The AlgEs work processively along their substrate, thus creating MG- or G-blocks by epimerizing every second residue (18–20). AlgE-type epimerases are the focus of this study.

AlgEs have been expressed and characterized from *Azotobacter vinelandii*, *A. chroococcum*, and *Pseudomonas syringae* (4, 21, 22). All AlgEs have the same modular structure consisting of combinations of catalytically active A-modules and carbohydrate-binding R-modules, which leads to the AlgEs having different substrate specificity and product profiles (15).

*A. vinelandii* encodes seven AlgEs (AlgE1-7), each consisting of one to two A-modules followed by one to four R-modules. All seven epimerases have different product profiles (4, 21). In this study, AlgE1 (147.2 kDa), AlgE4 (55.4 kDa), and AlgE6 (90.2 kDa) from *A. vinelandii* were used due to their difference in size, modular composition, and products formed. AlgE1 is a large epimerase composed of six modules in total and is able to create very long G-blocks (>100) (23–25). AlgE4 is the smallest epimerase consisting of only one A- and one R-module, and it creates MG-blocks (18, 26). In between these is AlgE6 consisting of one A- and three R-modules. AlgE6 also creates long G-blocks (~40 residues), although still shorter than AlgE1 (27–29).

The R-modules of AlgE4 and AlgE6 have been studied, showing that AlgE4R binds polyM stronger than all three AlgE6 R-modules combined, even though all are structurally similar (30). AlgE4R has a positively charged binding groove (31), which is shared to a lesser extent by AlgE6R_1_, but not AlgE6R_2_ and R_3_ (30). The R-modules of AlgE1 have not been studied to the same extent, except showing that removal of the R-modules reduces, but not abolishes, the activity of AlgE1 (24). Epimerases hold potential in industrial use for increasing the G-content of alginate, thereby improving its functionality and usability (15). One study has used AlgE1 for valorization of alginate in a process on a 20 L scale, increasing the amount of G from 44% to 76% (32).

Two problems with using enzymes industrially are that separating them from the product is not straightforward and that they cannot readily be reused for further epimerization. Both problems could be solved by immobilization of the enzymes. In this study, alginate epimerases were immobilized on *Bacillus subtilis* spores. Studies of immobilized cellulases (another group of processive and hydrolytic carbohydrate active enzymes) have shown that they retain their activity and processivity after immobilization (33). For example, an endoglucanase from *B. subtilis* was immobilized on a polymer matrix and retained its ability to hydrolyze cellulose, and the immobilized enzyme could be recycled at least five times while still being active (34). Immobilization can both increase and decrease the activity of an enzyme, but stability is usually enhanced (33). Furthermore, immobilization can change the enzyme conformation (35) and specificity (36). Other studies of immobilized non-processive carbohydrate-active enzymes include a glucose isomerase grafted onto carrageenan (37) and galactosyltransferase biotin-streptavidin beads (38). To the best of our knowledge, no processive, non-lytic enzyme has been immobilized by fusion to a bacterial spore so far.

Spores of *B. subtilis* are formed as a response to environmental stress and can be engineered to display heterologous enzymes on their surface. The gram-positive bacterium reacts to, e.g., nutrient starvation by forming the spore, a multilayered proteinaceous hull (39). During sporulation, the forespore is surrounded by a proteinaceous coat and cortex, formed by layer-specific proteins. The outermost layer, the crust, is made up of the crust proteins CotX, CotY, and CotZ, among others (40). Once spore formation is completed, the mother cell lyses, releasing the spore into the environment (41).

It encapsulates a copy of the cell’s DNA, protecting it from damage until environmental conditions become more favorable again, triggering the spore to germinate into a metabolically active cell.

Due to their stability, spores are easily separable and purifiable from a grown culture of *B. subtilis* by centrifugation (42). They offer a high physical resistance, for example, to wet and dry heat, UV radiation, and (to a certain extent) organic solvents, as well as to lysozyme (43). Combined with the fact that the spores themselves are metabolically dormant, many processes involving *B. subtilis* spores are generally recognized as safe (GRAS) by the FDA, and through the ease of genetic manipulation, they are an ideal vessel for surface display for a variety of enzymes (44, 45). This display technique, in which a native spore protein is used to anchor a protein of interest, is known as spore surface display.

A variety of spore proteins have been used in previous studies to anchor enzymes and antigens (45) to the spore surface. This is achieved by creating a fusion protein, which is connected to its anchoring protein through a linker and is expressed during sporulation. Due to the spores’ size and insolubility, they can be readily separated from liquid by centrifugation, offering easy purification of the immobilized fusion protein to a purity which is sufficient for a wide variety of applications. This also enables separation of the immobilized enzyme by centrifugation from a reaction mix after the reaction concludes, enabling reutilization (46).

Fusion proteins are frequently used to facilitate purification by combining the protein of interest with affinity tags (47), which allows column purification, or with another protein that aids in soluble expression (48). In most cases, this fusion partner is removed before continuing work with the purified protein for characterization and processing. Tobacco etch virus protease is a highly selective protease, which recognizes a seven amino acid long sequence and is commonly used in molecular biology to cleave fusion protein linkers (49). Zander and coworkers showed previously that protein displayed on the surface of *Paenibacillus polymyxa* can be cleaved off using TEV protease; however, no optimization of the conditions was performed, and no similar possibility has been reported for *B. subtilis* spores so far (50).

Our hypothesis is that immobilizing alginate epimerases on a spore surface leads to a resilient and recyclable system, while keeping the activity of the immobilized enzyme, allowing for easier and more efficient use of epimerases in industrial applications. Furthermore, cleaving the linker to the protein of interest could be used to compare enzyme activity on and off the spore surface. To investigate this, we produced *B. subtilis* spores displaying a variety of epimerase constructs and characterized their activity using NMR spectroscopy. This study contributes to better understanding of the impact of immobilization on processive, non-lytic enzymes, as well as expanding the knowledge of spore-immobilized enzymes.

## MATERIALS AND METHODS

Chemicals were purchased from Sigma-Aldrich (Darmstadt, Germany), VWR (Radnor, USA), or Roth (Karlsruhe, Germany). A list of ordering numbers can be found in the supplemental material (Table S1). Bovine serum albumin (BSA) and Tobacco etch virus protease were purchased from New England Biolabs (Ipswich, MA, USA).

### Transformation and strains

Replicative shuttle plasmids were used to display fusion proteins on the spore surface of *B. subtilis*. CotY served as the fusion partner and anchor protein, facilitating the integration of fusion proteins into the outermost spore layer (the crust) during sporulation. The fusion protein is under control of a sporulation-specific promoter (P*cotYZ*). Seamless Ligation Cloning Extract (51) was used for plasmid creation, and plasmids were transformed into *Escherichia coli* grown at 37°C in Lysogeny Broth (LB) media for maintenance. Transformed plasmids were confirmed by colony PCR and sequencing. *B. subtilis* was transformed using the natural competence of *B. subtilis* and a high copy shuttle plasmid as previously published by Karava et al. (52). Strain BS02003 is germination deficient, which keeps spores from developing into live cells again. All strains are described in Table 1.

**TABLE 1:** Relevant strains and their phenotypes^*a*^

Displayed enzyme	Strain	Short name	Relevant genotype	Strain background
AlgE4	BS29068	AlgE4-C-Rigid	Plasmid: CASC1 (*alg*E4-TEVs-Rigid-*cot*Y)	BS02003
	BS29097	AlgE4-N-Rigid	Plasmid: CASC1 (*cot*Y-Rigid-TEVs-*alg*E4)	BS02003
	BS29092	AlgE4-N-Flex	Plasmid: CASC1 (*cot*Y-Flex-TEVs-*alg*E4)	BS02003
	BS29093	AlgE4-C-Flex	Plasmid: CASC1 (*alg*E4-TEVs-Flex-*cot*Y)	BS02003
AlgE1	BS21014	AlgE1-C-Flex	Plasmid: CASC1 (*alg*E1-TEVs-Flex-*cot*Y)	BS02003
AlgE6	BS29085	AlgE6-C-Flex	Plasmid: CASC1 (*alg*E6-TEVs-Flex-*cot*Y)	BS02003
sfGFP	BS29079	GFP-N-Flex	Plasmid: CASC1 (*cot*Y-Flex-TEVs-*sfgfp*)	BS02003
	BS29080	GFP-N-Rigid	Plasmid: CASC1 (*cot*Y-Rigid-TEVs-*sfgfp*)	BS02003
	BS29082	GFP-C-Flex	Plasmid: CASC1 (*sfgfp*-TEVs-Flex-*cot*Y)	BS02003
	BS29083	GFP-C-Rigid	Plasmid: CASC1 (*sfgfp*-TEVs-Rigid-*cot*Y)	BS02003
–	BS02003	Control strain	Genomic: Δ*cwl*D Δ*sle*B::lox72 (germination deficient)	*Bacillus subtilis* KO7
	Plasmid CASC1	pCASCADEv1.0 (pMSE4)	ColE1-rep_origin (EC), KanR, Resolvase, RepE-rep_origin (BS), P*cot*YZ-promoter	

^*a*^ Strain BS02003 was transformed with the replicative plasmid CASC1. The relevant insert under control of the sporulation-specific PcotYZ promoter is given in parentheses for each strain. Flex, flexible linker; Rigid, semi-rigid linker. Linker sequences can be found in the supplemental material.

### Sporulation

Sporulation was initiated by nutrient starvation. *B. subtilis* strains were grown in LB with 5 g/L NaCl (LB5) media at 37°C and 225 rounds per minute (rpm) for 14 hr. For green fluorescent protein (GFP)-displaying strains, DSM media (53) supplemented with glucose to a final concentration of 0.5 g/L was used for sporulation. For sporulation of epimerase-displaying strains, a mix of 80% 2× Schaeffer’s glucose media (54) and 20% 500 mM 3-(*N*-morpholino)propanesulfonic acid (MOPS) buffer (pH 6.8) was used. Sporulation media was inoculated to a starting optical density at 600 nm (OD_600nm_) of 0.1 from the LB preculture. The culture was then incubated in a shaking incubator for 48 hr at 37°C and 225 rpm.

### Spore purification

After confirming sporulation by checking samples under the microscope for successful sporulation, the culture was harvested by centrifugation (3,600 times relative centrifugal force [RCF] for 15 min). Afterward, the pellet was resuspended in buffer (GFP-displaying spores: 100 mM NaCl, 50 mM Tris, pH 7.5; epimerase-displaying spores: 20 mM 4-(2-hydroxyethyl)-1-piperazineethanesulfonic acid (HEPES), 75 mM NaCl, 2.5 mM CaCl_2_, pH 6.9) containing 0.5 mg/mL of chicken egg white lysozyme. Ten milliliters of this lysis solution was used per 50 mL of spore production culture. The resuspended pellet was incubated for 1 hr, and lysis of all remaining cells was verified by microscopy. The spores were then washed twice by centrifugation (2,500 RCF, 15 min) and resuspension in fresh buffer, followed by a final centrifugation (2,300 RCF) and resuspension in a small volume of buffer (around 2 mL/200 mL of original culture). OD_600nm_ was then adjusted to the required density, and spores were stored at 4°C.

### TEV cleavage GFP-displaying spores

To compare the efficiency of TEV cleavage on different linker and display directions, two N- and two C-terminal fusions between CotY and the GFP variant sfGFP (superfolder GFP) were created with a flexible glycine-rich linker (GGGGS)_2_, as well as a more rigid α-helix forming linker (EAAAK)_3_. A final reaction mix of 50 mM Tris-HCl pH 7.5, 1 mM dithiothreitol (DTT), 0.5 mM ethylenediaminetetraacetic acid, and 200 units per mL (U/mL) TEV protease was used. The 150 µL reaction mixture in 1.5 mL microcentrifuge tubes, containing spores at OD_600nm_ of 1, was incubated for 18 h at 1,400 rpm in a benchtop thermoshaker. Afterward, the mixture was centrifuged at 10,000 RCF for 10 min and the supernatant taken off and centrifuged again. Fifteen microliters was then transferred to a 384-well plate, and fluorescence was measured in a PHERAstar FSX plate reader (BMG Labtech; 485 nm/520 nm). For each condition, six replicates were measured.

### TEV cleavage optimization

The N-terminally linked GFP-displaying strain with a flexible linker GFP-N-Flex (Table 1) was used for optimizing the reaction conditions. For the preparation of the sample with the highest bovine serum albumin (0.5%) concentration and TEV protease concentration (800 U/mL), the following protocol was used: in a 1.5 mL microcentrifuge tube, 20 µL of purified spores at twice the target OD_600nm_ is mixed with 10 µL BSA solution (20 mg/mL). Afterward, 4 µL 500 mM Tris-HCl pH 7.5, 0.16 µL 500 mM DTT solution, 2.8 µL ultrapure water (UPW), and 3.2 µL TEV (10,000 U/mL) are added. For lower concentrations of TEV or BSA, volume was substituted with UPW. The solution is mixed by pipetting up and down and incubated 18 h at 30°C at 1,400 rpm in a benchtop thermoshaker. Afterward, samples were centrifuged at 10,000 RCF for 10 min, and 35 µL sample was transferred into a new tube and centrifuged again. Twenty-eight microliters of sample per tube was finally transferred into a 384-well microplate. Fluorescence was measured in a PHERAstar FSX plate reader (BMG Labtech; 485 nm/520 nm).

### TEV cleavage of epimerase-displaying spores

Cleavage of AlgE4-displaying spores was performed in HEPES buffer (20 mM HEPES, 75 mM NaCl, 2.5 mM CaCl_2_, pH 6.9) also used for later epimerization. A final spore OD_600nm_ of 10 was used, together with 0.2% BSA and a TEV concentration of 400 U/mL. DTT concentration in the reaction mix was 2 mM. The reaction was incubated at 30°C for 18 h at 1.400 rpm on a benchtop thermoshaker. Afterward, supernatant and spores were separated by centrifugation as described earlier. The spores were resuspended in HEPES buffer to a final volume of 200 µL, and both supernatant and resuspended spores were treated as separate samples for an alginate epimerization performed afterward.

### Alginate substrates

PolyM was produced from an AlgG-deficient strain of *Pseudomonas fluorescens* resulting in a substrate of F_M_ = 1.00 (55). The polyM was acid hydrolyzed to a number average degree of polymerization around DP = 70–100 (56) before epimerization. ^13^C-1-labeled polyM was produced from the same *P. fluorescens* strain using ^13^C-1-labeled D-fructose and depolymerized using the same acid hydrolysis method. Producing polyM with this method results in the polyM not being acetylated, and therefore it is chemically identical to the polyM found in alginate from seaweed.

### Alginate epimerization

Experiments were carried out in HEPES buffer (20 mM HEPES, 75 mM NaCl, 2.5 mM CaCl_2_, pH 6.9). Purified spores were adjusted to OD_600nm_ of 10 unless specified otherwise. Two hundred microliters of spore suspension was mixed with 426 µL of a 10 mg/mL polyM solution in a 1.5 mL microcentrifuge tube (final OD_600nm_ of 3.19). The sample was incubated in a benchtop shaker at 1,000 rpm of shaking. After incubation, the sample was centrifuged at 10,000 RCF for 5 min, and the supernatant taken off and transferred into a new tube, which was again centrifuged at 10,000 RCF for 5 min. The supernatant was then transferred to a new tube again and freeze-dried for later NMR analysis.

### Spore recycling

Epimerization was carried out as described earlier at either 37°C or 50°C for 12 h. Afterward, the spore alginate mixture was centrifuged, and the supernatant was taken off. The pellet was then resuspended in 426 µL alginate solution (polyM; 10 mg/mL) and 195 µL of HEPES buffer. The 12 h incubation was then repeated with the fresh substrate for a total of five times. The supernatant was treated and analyzed as described earlier.

### Product characterization using NMR

After the epimerization reaction, the reaction mixture was freeze-dried and redissolved in D_2_O (99.9%, Sigma-Aldrich) with 0.05% 3-(trimethylsilyl)-propionic-2,2,3,3-d_4_ acid sodium salt as an internal reference and 0.01 M triethylene-tetraamine-hexaacetate as a calcium chelator. A 1D ^1^H spectrum was run of each sample at 83°C with 64 scans and a spectral width of 10 ppm.

All NMR spectra at 83°C were recorded on a BRUKER NEO 600 MHz instrument (Bruker BioSpin AG, Fälladen, Switzerland) equipped with a 5 mm iProbe TBO preheated to 83°C.

### Time-resolved NMR for reaction analysis

Time-resolved analysis was performed by dissolving ^13^C-1-labeled polyM (DP ~70, 10 mg/mL) in a buffer of 5 mM MOPS pH 6.9, 2.5 mM CaCl_2_, and 75 mM NaCl in D_2_O (99.9%, Sigma-Aldrich) and transferring 160 µL to an NMR tube. The sample was preheated to 50°C in the NMR magnet, and 1D ^1^H and ^13^C spectra were recorded. Then, 80 µL of spores (OD_600nm_ of 20) was added, and the sample was mixed by inverting the tube three times. The sample was put back in the magnet, and a pseudo-2D spectrum was recorded by recording a ^13^C 1D spectrum every 10 min for 15 h and 20 min. Afterward, a ^1^H-^13^C HSQC was recorded. ^1^H-signals were internally referenced to the water signal, and ^13^C signals were indirectly referenced to the water signal based on absolute frequency ratios (57). All time-resolved NMR spectra were recorded on a Bruker Avance III HD 800 MHz spectrometer using a 5 mm Z-gradient CP-TCl (H/C/N) cryogenic probe.

The 800 MHz and 600 MHz NMR spectrometers were located at the NV-NMR-Center/Norwegian University of Science and Technology. All spectra were recorded using TopSpin 3.6 pl 7 or 4.0.8 software (Bruker BioSpin) and processed and analyzed using TopSpin 4.3.0 or 4.4.0 software (Bruker BioSpin).

## RESULTS

### AlgE4 is active on the spore surface and creates MG-blocks

The goal of this study was to produce *B. subtilis* spores with active alginate epimerases on the spore surface as a way of immobilizing the enzymes to create a resilient system for easy enzyme production, purification, and reuse.

A library of fusion constructs was generated to display AlgE4 in two different orientations (N- and C-terminally bound to CotY). For each orientation, a flexible poly-glycine linker variant, as well as a variant with a more rigid α-helical linker, was created, resulting in four strains. All variants included a TEV cleavage site between the linker and the alginate epimerase. The fusion constructs were screened for enzymatic activity by incubating the purified spores with alginate at 24°C for 24 h. Afterward, the sample was analyzed by NMR spectroscopy.

**Fig 1:**
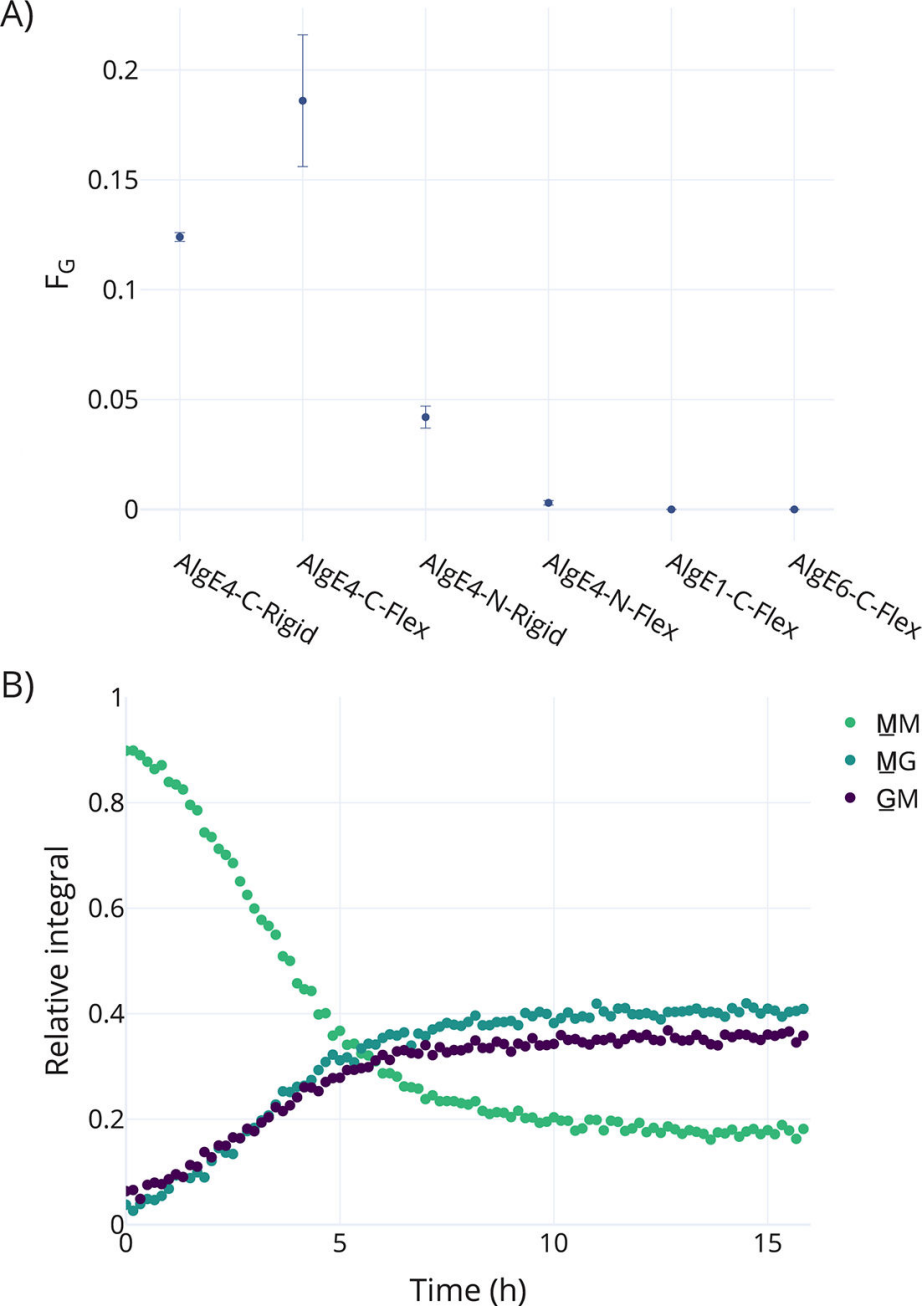
(**A**) Reaction of different spore strains (OD_600nm_ of 3.2) with polyM (DP ~70, 6.39 mg/mL) at 24°C for 24 h. The *y*-axis shows the relative fraction of G-residues in the reaction product (F_G_). All strains showing activity display AlgE4 (*n* = 3). (**B**) Integrals from time-resolved NMR spectra of reaction between AlgE4-C-Rigid (OD_600nm_ of 6.7) and ^13^C-1-labeled polyM (DP ~70, 6.67 mg/mL). The reaction was run for 15 h and 20 min at 50°C with a 1D ^13^C spectrum recorded every 10 min. The three anomeric signals were integrated, and the annotation shows the corresponding residue and its neighbor toward the reducing end (MM, M with M as neighbor; MG, M with G as a neighbor; GM, G with M as a neighbor). No signals for G-blocks could be observed. The reaction was run at 50°C to avoid overlap of key signals with the water signal. After the reaction was finished, a ^1^H-^13^C HSQC was recorded to confirm signal assignment (Fig. S1).

All four AlgE4-containing strains displayed enzymatic activity but notably more for the two C-terminal-linked AlgE4 strains. One of the strains containing N-terminally linked enzyme showed activity barely detectable compared to the noise with a fraction of G-residues (F_G_) = 0.003 ± 0.001 (AlgE4-N-Flex) (Fig. 1A). Two strains displaying C-terminally linked AlgE1 (AlgE1-C-Flex) and AlgE6 (AlgE6-C-Flex) were investigated in the same manner. Neither of these strains showed any activity (Fig. 1A).

The strain AlgE4-C-Rigid was chosen for further experiments. To understand if immobilization of AlgE4 impacts its mode of action, the reaction of AlgE4-C-Rigid with polyM was investigated using time-resolved NMR and ^13^C-1-labeled PolyM (Fig. 1B). Inspection of the NMR tube after 15 hr in the NMR magnet showed that the spores (OD_600nm_ of 6.7) were still in suspension. This is likely due to convection within the NMR tube that kept spores in suspension and facilitated continuous mixing in the tube. At temperatures around 37°C, key NMR signals were obscured by overlap with the water signal; therefore, 50°C was chosen. The initial ~5 min of the reaction was not observed due to the time between mixing the reagents and acquiring the first NMR spectrum. The curves shown in Fig. 1B start out nonlinear. This might be due to a slight signal overlap between the MM and GM signals in the first four spectra. MG-blocks were formed, but no G-blocks, and the total amount of G formed was ~40%.

AlgE4 epimerizes polyM to polyMG, with a theoretical maximum F_G_ of 0.5 for complete epimerization. AlgE4 does not normally form G-blocks; therefore, it was to be expected that no G-blocks were formed. Spore concentrations were optimized to enable observable epimerization without reaching full conversion of polyM to polyMG (theoretical maximum F_G_ of 0.5). The intent was that both increases and decreases in epimerization levels between different strains and treatments could be detected.

In the time-resolved NMR analysis of the reaction of AlgE4-C-Rigid and polyM, the G product formation is around 40%, which is as high as can be expected for this length of substrate. The time-resolved NMR experiment was run with an OD_600nm_ of 6.7 at 50°C, whereas the bench top epimerization experiments used OD_600nm_ of 3.2 at 24°C in the reaction solution. This shows that it is possible to obtain the product amount that is desired by changing the spore OD_600nm_ and temperature.

### AlgE4 on the spore surface can be recycled

**Fig 2:**
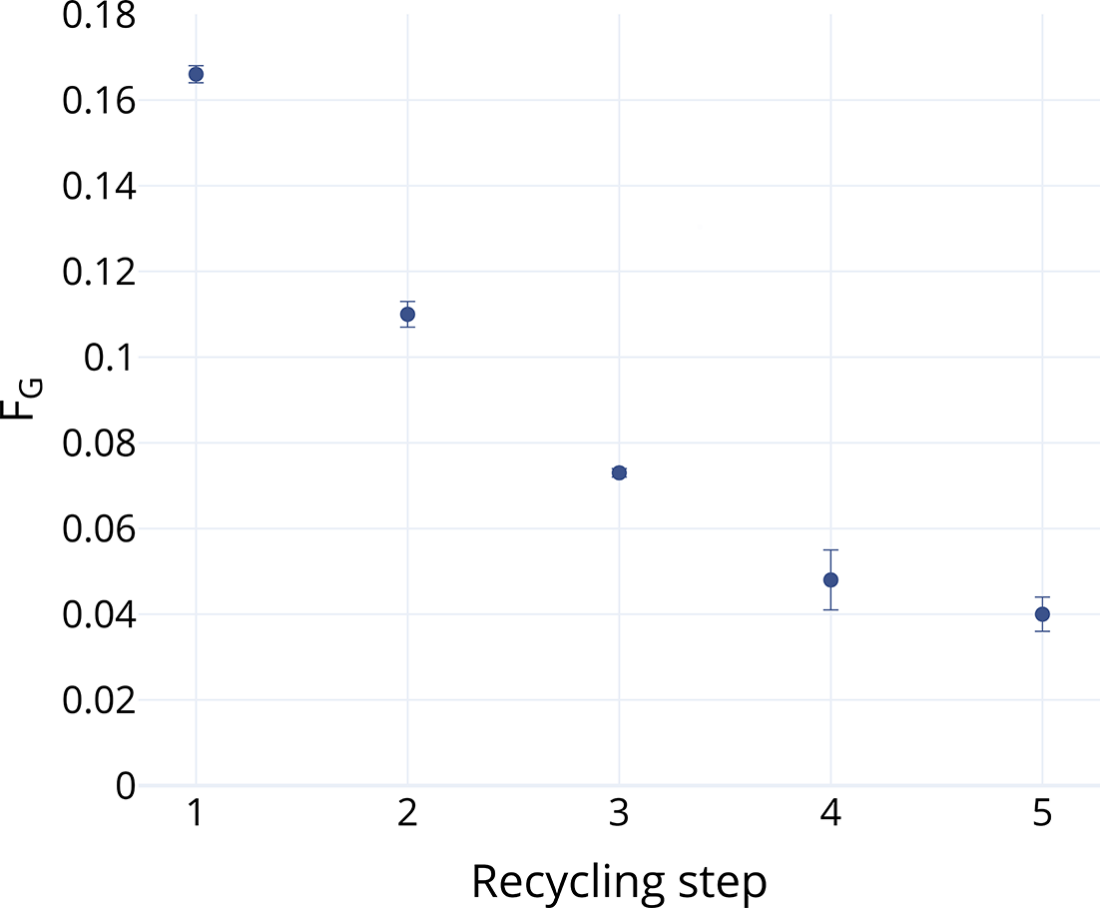
Reaction between AlgE4-C-Rigid (OD_600nm_ of 3.2) and polyM (6.39 mg/mL), where the same spores are recycled for each 12 h reaction run. The *y*-axis shows the fraction of G-residues (F_G_) in the product. The reaction was run at 37°C, the optimal reaction temperature for AlgE4 (18) (*n* = 3).

To assess the recyclability of the spore-displayed epimerases, five rounds of epimerization at 37°C for 12 h each using the same spore batch of AlgE4-C-Rigid were performed. The chosen temperature has previously been shown to be the optimal reaction temperature for AlgE4 (18). In the first reaction, F_G_ = 0.16 was achieved, while in the second F_G_ = 0.11 was achieved, corresponding to a 34% reduction in activity compared to the first reaction (Fig. 2). Nevertheless, the spores were still active in five reaction cycles, with the activity having dropped to 24% in the fifth round with F_G_ = 0.04 (Fig. 2), which is comparable to other preliminary studies on spore recycling (58, 59).

The decline in activity after each recycling step could be attributed to incomplete recovery of spores during centrifugation. But retaining a significant proportion of activity after only one simple purification step shows that this system has potential to reduce enzyme costs in industrial applications (60) after optimizing the recovery process.

Recycling was also tried at elevated temperatures of 50°C to investigate long-term stability of the displayed enzyme at an elevated temperature, but activity dropped rapidly after the first spore recycling, with no detectable epimerization in cycle four (Fig. S2).

### TEV cleavage system optimized with GFP protein

To assess whether spore-displayed fusion proteins can be cleaved on the spore surface, a library of strains displaying a CotY-sfGFP fusion protein was generated, enabling monitoring of linker cleavage via a fast fluorescence-based measurement. The sfGFP was genetically fused to the N-terminus, as well as the C-terminus of the crust protein CotY. For each orientation, a flexible glycine-rich linker (GGGGS)_2_, as well as a more rigid α-helix forming linker (EAAAK)_3_ (61), was tested. After incubation for 18 h at 30°C, the spores were removed by two consecutive centrifugation steps, and fluorescence was measured in the supernatant. No TEV was added to the control samples. Six replicates were tested for each condition. For all variants, there was a significant difference between TEV-treated and untreated samples (between 19 to fivefold), except for the control strain, which showed very low fluorescence in both conditions. The supernatant of spores displaying sfGFP fused to the C-terminus of CotY exhibited a significantly higher fluorescence in the supernatant compared to N-terminal fusions (104,000 and 49,000 RFU vs 14,000 and 5,000 RFU), indicating a greater concentration of free sfGFP in the supernatant. For both fusion orientations, the rigid linker variants released less sfGFP into solution compared to the flexible linker variant.

The strain GFP-N-Flex was chosen to further optimize the cleavage conditions. The influence of varying TEV and BSA concentrations was tested, as well as the influence of different spore ODs. Three levels of each variable were tested in a full factorial layout (Fig. 2B). A spore OD_600nm_ of 1 showed a quarter to a fifth of fluorescence when compared to an OD_600nm_ of 5 (Fig. S3). Addition of 0.2% BSA to the spores prior to starting cleavage has a positive effect on the observed fluorescence levels. Higher BSA concentrations of 0.5% in the reaction mix led to lower measured fluorescence in the supernatant for OD_600nm_ of 1 and 5 but have a positive effect for OD_600nm_ of 10 spore reaction mixes with TEV concentrations of 400 and 800 U/mL. While doubling the TEV concentration shows a strong effect at OD_600nm_ of 10 when going from 200 to 400 U/mL, a further increase to 800 U/mL does not lead to a proportional increase in measured fluorescence.

This suggests that the reaction conditions approached the maximum achievable release of sfGFP from the surface. Since spores readily adsorb proteins (62), one possible explanation for the effect of premixing the spores with BSA is a blockage of sites where TEV or cleaved sfGFP could get adsorbed onto the spores’ surface, rendering the TEV effectively inactive or sequestering the sfGFP from the supernatant.

### AlgE4 was cleaved off the spore surface

To determine whether an enzyme can be cleaved from the spore surface after purification, multiple AlgE displaying strains were constructed with either a flexible or rigid linker (the same two linkers as used for the sfGFP spores) and a TEV recognition site fused to the N- or C-terminus of AlgE4. This will provide insights into how different fusion forms affect enzyme activity and cleavage efficiency.

The four spore variants displaying AlgE4 were treated with TEV protease (400 U/mL) in the epimerization buffer. After 18 h of incubation at 30°C, spores and supernatant were separated by centrifugation. The spores were resuspended in fresh buffer. Both samples (spores and supernatant), as well as samples not treated with TEV protease, were mixed with 430 µL of a 10 mg/mL alginate solution, incubated (12 h; 37°C, 1,000 rpm) and analyzed by NMR.

**Fig 3:**
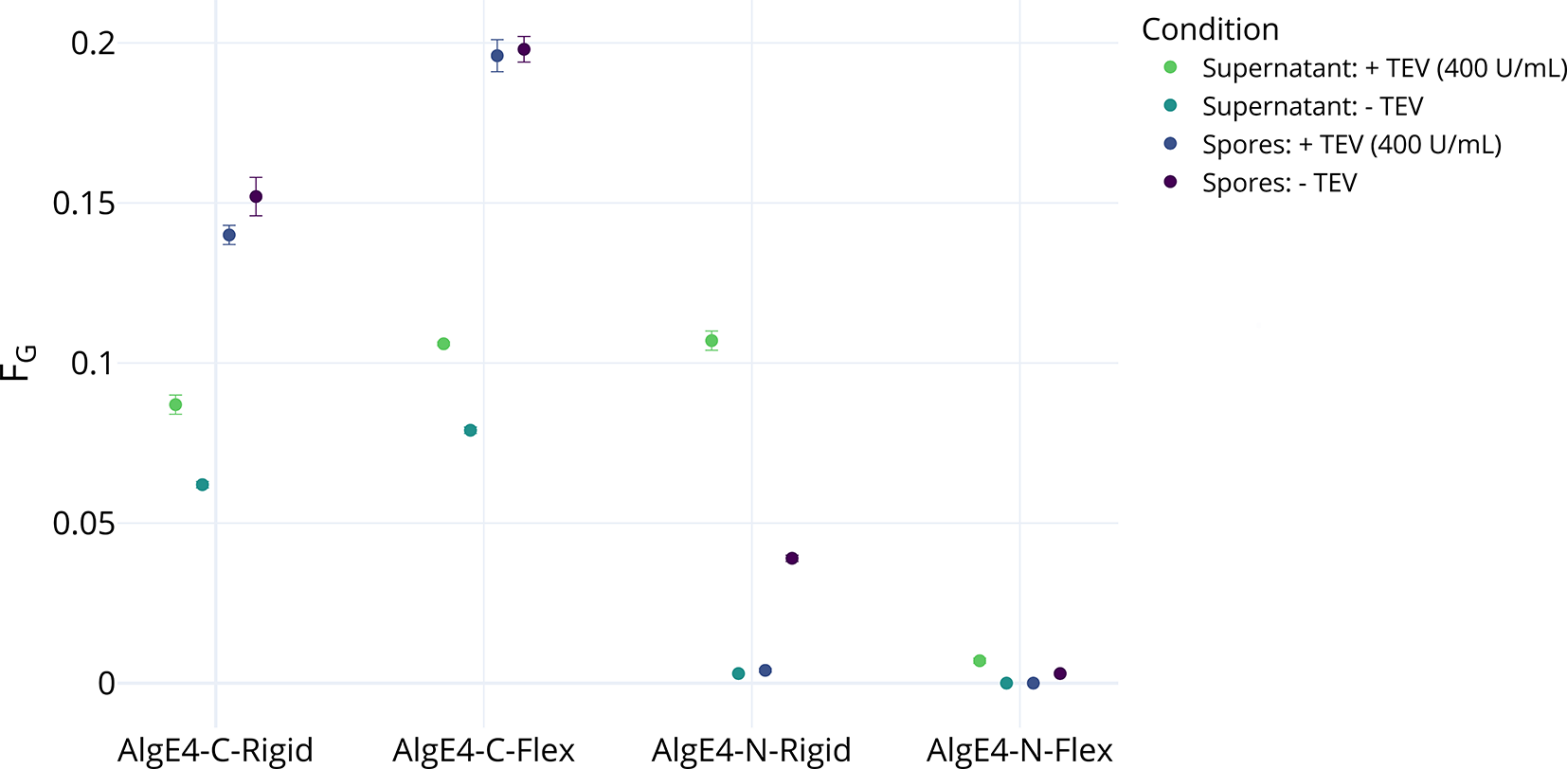
Effect of TEV treatment on AlgE4-displaying spores. Each spore strain was either treated with TEV (400 U/mL) or no TEV was added, but in either case, the spores were incubated for 18 h at 1,400 rpm, using the cleavage conditions determined using sfGFP spores. After incubation, the supernatant and spores were separated and reacted with polyM for 12 h at 37°C. For all four strains, the supernatant with the highest activity was the one where the spores were treated with TEV, and the spore pellet with the highest activity was the one where the spores were untreated (*n* = 3).

For all four strains, the epimerization activity in the supernatant of samples treated with TEV protease was higher than the untreated supernatant samples (Fig. 3). No change in the mode of action was observed when comparing cleaved off and spore-bound enzyme, with both variants epimerizing MM-blocks into MG-blocks. The background activity for untreated supernatant samples was comparatively high for spores on which the epimerase is bound to the C-terminus of AlgE4 (N-terminus of CotY), reaching 72%–75% of the activity of the treated supernatant samples.

For epimerases bound via the N-terminus of AlgE4, the difference between supernatant samples was significantly higher. For AlgE4-N-Rigid, the untreated supernatant samples only reached around 3% of the epimerization rates of the treated samples. Epimerization activity was very low for the spores as well as the supernatant of AlgE4-N-Flex, with no detectable activity in untreated supernatant samples and treated spore samples. The activity observed in the supernatant for both latter strains was higher in the supernatant than at the spores’ surface, with a strong decrease in measured on-spore activity for treated samples, indicating that a large portion of active and available AlgE4 was cleaved off.

Epimerization in the supernatant was disproportionately increased after TEV cleavage in comparison to the reduction of epimerization by the treated spores themselves in all samples. This effect was particularly pronounced in the N-terminally fused AlgE4-N-Rigid and AlgE4-N-Flex, where the increase in epimerization activity between treated and untreated supernatant samples was more than double the decrease in activity observed between untreated and treated spore samples. While an F_G_ of 0.107 was reached in the supernatant of AlgE4-N-Rigid, only F_G_ = 0.039 was achieved by the untreated spores. For AlgE4-N-Flex, 0.7% of M-residues converted into G (F_G_ = 0.007) in the cleaved supernatant samples compared to F_G_ = 0.003 in the untreated spores.

For both strains linking AlgE4 via the C-terminus, the epimerized fraction of product in the supernatant was around 0.026 higher than in the untreated supernatant. Epimerization fractions achieved by the resuspended spores dropped by 0.012 (AlgE4-C-Rigid) and 0.002 (AlgE4-C-Flex) when the spores had been TEV treated. This might indicate that either the activity of free alginate epimerase is significantly higher compared to spore-bound epimerase or that not all epimerases on the spore surface are accessible for reaction but become accessible to the substrate as TEV cleavage progresses.

The lower efficiency in cleavage possibly stems from the different termini to which the epimerase is fused on the spore. A possible explanation could be that the cleavage site in the linker to the C-terminally bound epimerases is less accessible for TEV protease. Their spores do, however, at the same time display a higher degree of epimerization when compared to strains in which AlgE4 is linked on the N-terminus. Differences in the amount of expressed and/or displayed enzyme or the substrate accessibility could also be responsible for the observed differences in activity.

**Fig 4:**
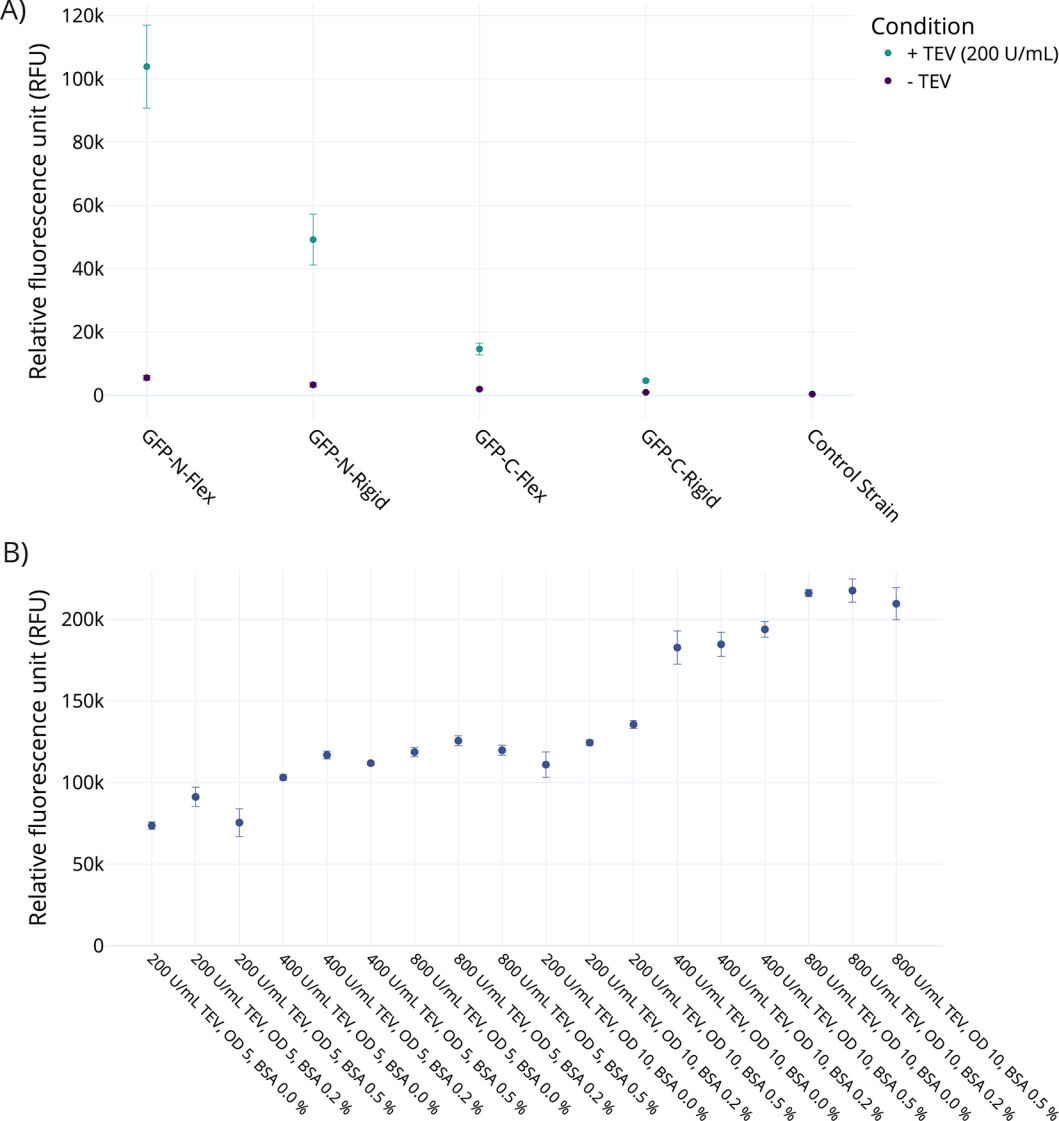
Measured sfGFP fluorescence in the supernatant after treatment of sfGFP displaying spores with TEV. Cleavage was performed for 18 h at 30°C, spores were separated by centrifugation, and fluorescence of the supernatant was measured. (**A**) Supernatant fluorescence for different strains and a control strain with either TEV added (+) or not added (−); (OD_600nm_ of 1; *n* = 6). (**B**) Varied amounts of TEV, BSA, and spores to determine the optimal cleavage conditions of GFP-N-Flex. Increasing BSA concentrations seems to have a small positive impact on the reactivity. Increasing the amount of TEV from 200 U/mL to 400 U/mL has a large impact on fluorescence, whereas doubling the TEV amount again from 400 U/mL to 800 U/mL has a smaller impact. Furthermore, increasing spore concentration from OD_600nm_ of 5 to OD_600nm_ of 10 resulted in significantly higher fluorescence (*n* = 3).

The observed high epimerization in untreated supernatant samples of C-terminal AlgE4-C-Rigid and AlgE4-C-Flex could be due to protein detaching from the crust during the 18 hr of shaking. Some indication for this can be found in the earlier measurements of free sfGFP after shaking (Fig. 4A), where C-terminally bound sfGFP (bound to the N-terminus of CotY) also came off the spore surface in the control reactions (w/o TEV) at a higher ratio compared to N-terminally fused sfGFP (fused to the C-terminus of CotY). The low background activity in N-terminally bound AlgE4 strains could also hint that the fusion proteins’ position in the spore’s crust is more secured and therefore less likely to detach during simple shaking. However, while much higher fluorescence was observed for N-terminal fusions of GFP, the N-terminal AlgE4 fusions showed a lower degree of epimerization activity compared to their C-terminal counterparts, which could be due to misfolding or the enzyme being truncated. It is possible that the display efficiency is an effect which is dependent in large parts on the respective displayed protein; however, elucidating the exact factors at play during expression and localization of different fusion proteins during spore formation will require extensive research.

TEV cleavage was also attempted for the inactive AlgE1 and AlgE6 displaying strains to see if activity could be gained by freeing the enzyme from the spore. No activity was detected for either strain after cleavage. This could indicate that the reasons for the inactivity of AlgE1 and AlgE6 are not them being immobilized and sterically hindered but rather issues with misfolding or expression.

AlgE1 and AlgE6 are both much larger enzymes than AlgE4. AlgE4 consists of one A-module and one R-module, and AlgE6 has two R-modules more (4, 21). AlgE1 has two A-modules and four R-modules, making it the largest enzyme tested. Possibly, the size of the displayed protein impacts whether the protein can be correctly produced or not, since AlgE4 spores showed activity, but AlgE1 and AlgE6 spores did not.

AlgE4 is a processive enzyme active on a polysaccharide, and it is endo-active and non-lytic (20). All these factors could negatively impact the enzyme activity for an immobilized AlgE4, because of steric hindrance, limited accessibility, and low mobility, all of which are more problematic for an endo-active, processive polysaccharide-active enzyme. Yet, AlgE4 immobilized on the spore was active, and immobilization under the conditions investigated here did not impact the mode of action of the epimerase.

The most active AlgE4 strains contained C-terminally linked AlgE4, which means that AlgE4 is linked through its R-module. The C-terminus of the AlgE4 R-module contains a long flexible region of 22 amino acid residues (31), so when AlgE4 is linked through its C-terminal, the total length of the flexible region becomes longer than when it is linked through its N-terminal. This could explain the increased activity of C-terminally linked AlgE4 compared to N-terminally linked AlgE4. Simply, the enzymes having more freedom and being able to extend further from the spore surface might make a larger number of epimerases accessible for reaction.

The spore surface of *B. subtilis* spores is negatively charged (63). Alginate is also negatively charged (64), which is an argument for there not being any ionic binding between alginate and the spores. Had the spore surface been positively charged, possibly alginate would have been interacting more with the spore surface and not been as available for epimerization. Most of the surface of the alginate epimerases is also negatively charged, except for a positive binding groove (30, 31, 65), which could also be helping in keeping the epimerase further from the spore surface and therefore more accessible for reaction.

We achieved running an epimerase reaction with spore-displayed AlgE4 in an NMR tube while recording a time-resolved NMR spectrum. To our knowledge, this is the first published example of running a reaction with a *B. subtilis* spore in an NMR tube. The spores were thought to have stayed suspended because of heat convection inside the NMR tube. Shimming (homogeneity of the magnetic field) and recording spectra with the 800 MHz instrument were not hampered by the presence of spores or the viscosity of the reaction fluid. This opens up the possibility for future studies on the mode of action of immobilized enzymes.

### Conclusion and outlook

In this study, we aimed to characterize a processive, non-lytic enzyme immobilized on the spore surface of *Bacillus subtilis*. Four different alginate epimerases were tested. AlgE1 and AlgE6 do not show activity when immobilized on the surface of *B. subtilis* spores. However, AlgE4 immobilized on the spore surface by fusion to the crust protein CotY is active. The epimerase shows a higher degree of activity when fused via its C-terminus. When linked with a semi-rigid linker, the enzyme retains 24% of its initial activity after recycling the spore-displayed enzyme five times. This, together with the ability to tune the degree of epimerization by adjusting the amount of spores in the process, shows the potential of spores for the epimerization of alginate.

We further showed that spore-displayed proteins can be cleaved off the spore surface by use of TEV protease, enabling their controlled release after purification. For AlgE4, the release from the spore surface leads to an increase in observed activity. To broaden the range of processing options, it would be interesting to investigate if this is also the case with other proteases or self-cleaving linkers. This could offer increased cleavage efficiency for immobilizations on the C-terminus of AlgE4.

Quantification of enzymes bound on the spore surface has so far presented a major challenge, usually tackled by using specific antibodies against the protein of interest (66). We showed that the degree of activity of the spore-bound enzyme can be different from that of the free enzyme, depending on orientation and linker.

Cleaving off the displayed enzyme and measuring its activity will allow for a better understanding and comparison of the activity of bound and freed enzyme. It could also allow for comparing it with the activity of conventionally expressed enzyme in future works in the field.

This study has shown a processive and non-lytic enzyme immobilized on *B. subtilis* spores, which remains active and allows for efficient recycling and easy purification of the spore-enzyme system. This approach, combined with the scalable production and isolation of spores, presents promising opportunities for industrial applications of alginate epimerases immobilized on *B. subtilis* spores.

## Data Availability

Data are available in a Zenodo repository under the following DOI: 10.5281/zenodo.14243652.

## SUPPLEMENTAL MATERIAL

Supplemental material aem.00298-25-s0001.pdf Figures S1 to S3; Tables S1 and S2.

ASM does not own the copyrights to Supplemental Material that may be linked to, or accessed through, an article. The authors have granted ASM a non-exclusive, world-wide license to publish the Supplemental Material files. Please contact the corresponding author directly for reuse.

## References

[1] Linker A, Jones RS. 1964. A polysaccharide resembling alginic acid from a Pseudomonas micro-organism. Nature 204:187–188. doi:10.1038/204187a0 14222269

[2] Haug A, Myklestad S, Larsen B, Smidsrød O, Eriksson G, Blinc R, Paušak S, Ehrenberg L, Dumanović J. 1967. Correlation between chemical structure and physical properties of alginates. Acta Chem Scand 21:768–778. doi:10.3891/acta.chem.scand.21-0768

[3] Chitnis CE, Ohman DE. 1990. Cloning of Pseudomonas aeruginosa algG, which controls alginate structure. J Bacteriol 172:2894–2900. doi:10.1128/jb.172.6.2894-2900.1990 2160929 PMC209086

[4] Ertesvåg H, Høidal HK, Hals IK, Rian A, Doseth B, Valla S. 1995. A family of modular type mannuronan C-5-epimerase genes controls alginate structure in Azotobacter vinelandii. Mol Microbiol 16:719–731. doi:10.1111/j.1365-2958.1995.tb02433.x 7476166

[5] Tøndervik A, Aarstad OA, Aune R, Maleki S, Rye PD, Dessen A, Skjåk-Bræk G, Sletta H. 2020. Exploiting mannuronan C-5 epimerases in commercial alginate production. Mar Drugs 18:565–565. doi:10.3390/md18110565 33218095 PMC7698916

[6] Skjåk-Bræk G, Donati I, Paoletti S. 2015. Alginate hydrogels: properties and applications. 2nd ed. Jenny Stanford Publishing.

[7] Bennett JP, Robinson LF, Gomez LD. 2023. Valorisation strategies for brown seaweed biomass production in a European context. Algal Res 75:103248. doi:10.1016/j.algal.2023.103248

[8] Drummond DW, Hirst EL, Percival E. 1962. 232. The constitution of alginic acid. J Chem Soc 37:1208. doi:10.1039/jr9620001208

[9] Atkins EDT, Mackie W, Smolko EE. 1970. Crystalline structures of alginic acids. Nature 225:626–628. doi:10.1038/225626a0 16056655

[10] Grant GT, Morris ER, Rees DA, Smith PJC, Thom D. 1973. Biological interactions between polysaccharides and divalent cations: the egg-box model. FEBS Lett 32:195–198. doi:10.1016/0014-5793(73)80770-7

[11] Ertesvåg H, Valla S. 1998. Biosynthesis and applications of alginates. Polym Degrad Stab 59:85–91. doi:10.1016/S0141-3910(97)00179-1

[12] Lee KY, Mooney DJ. 2012. Alginate: properties and biomedical applications. Prog Polym Sci 37:106–126. doi:10.1016/j.progpolymsci.2011.06.003 22125349 PMC3223967

[13] Lin T-Y, Hassid WZ. 1966. Pathway of alginic acid synthesis in the marine brown alga, Fucus gardneri Silva. J Biol Chem 241:5284–5297. doi:10.1016/S0021-9258(18)96429-X 5954796

[14] Haug A, Larsen B. 1969. Biosynthesis of alginate. Epimerisation of d-mannuronic to l-guluronic acid residues in the polymer chain. Biochim Biophys Acta 192:557–559. doi:10.1016/0304-4165(69)90414-0 5368261

[15] Petersen AB, Tøndervik A, Gaardløs M, Ertesvåg H, Sletta H, Aachmann FL. 2023. Mannuronate C-5 epimerases and their use in alginate modification. Essays Biochem 67:615–627. doi:10.1042/EBC20220151 36876890

[16] Franklin MJ, Chitnis CE, Gacesa P, Sonesson A, White DC, Ohman DE. 1994. Pseudomonas aeruginosa AlgG is a polymer level alginate C5-mannuronan epimerase. J Bacteriol 176:1821–1830. doi:10.1128/jb.176.7.1821-1830.1994 8144447 PMC205283

[17] Rehm BH, Ertesvåg H, Valla S. 1996. A new Azotobacter vinelandii mannuronan C-5-epimerase gene (algG) is part of an alg gene cluster physically organized in a manner similar to that in Pseudomonas aeruginosa. J Bacteriol 178:5884–5889. doi:10.1128/jb.178.20.5884-5889.1996 8830682 PMC178442

[18] Høidal HK, Ertesvåg H, Skjåk-Braek G, Stokke BT, Valla S. 1999. The recombinant Azotobacter vinelandii mannuronan C-5-epimerase AlgE4 epimerizes alginate by a nonrandom attack mechanism. J Biol Chem 274:12316–12322. doi:10.1074/jbc.274.18.12316 10212201

[19] Hartmann M, Holm OB, Johansen GAB, Skjåk-Braek G, Stokke BT. 2002. Mode of action of recombinant Azotobacter vinelandii mannuronan C-5 epimerases AlgE2 and AlgE4. Biopolymers 63:77–88. doi:10.1002/bip.10017 11786996

[20] Campa C, Holtan S, Nilsen N, Bjerkan TM, Stokke BT, Skjåk-Braek G. 2004. Biochemical analysis of the processive mechanism for epimerization of alginate by mannuronan C-5 epimerase AlgE4. Biochem J 381:155–164. doi:10.1042/BJ20031265 15032753 PMC1133773

[21] Svanem BIG, Skjåk-Bræk G, Ertesvåg H, Valla S. 1999. Cloning and expression of three new Aazotobacter vinelandii genes closely related to a previously described gene family encoding mannuronan C-5-epimerases. J Bacteriol 181:68–77. doi:10.1128/JB.181.1.68-77.1999 9864314 PMC103533

[22] Gawin A, Tietze L, Aarstad OA, Aachmann FL, Brautaset T, Ertesvåg H. 2020. Functional characterization of three Azotobacter chroococcum alginate-modifying enzymes related to the Azotobacter vinelandii AlgE mannuronan C-5-epimerase family. Sci Rep 10:12470. doi:10.1038/s41598-020-68789-3 32719381 PMC7385640

[23] Ertesvåg H, Høidal HK, Skjåk-Braek G, Valla S. 1998. The Azotobacter vinelandii mannuronan C-5-epimerase AlgE1 consists of two separate catalytic domains. J Biol Chem 273:30927–30932. doi:10.1074/jbc.273.47.30927 9812987

[24] Ertesvåg H, Valla S. 1999. The A modules of the Azotobacter vinelandii mannuronan-C-5-epimerase AlgE1 are sufficient for both epimerization and binding of Ca^2+^. J Bacteriol 181:3033–3038. doi:10.1128/JB.181.10.3033-3038.1999 10322003 PMC93757

[25] Aarstad OA, Stanisci A, Sætrom GI, Tøndervik A, Sletta H, Aachmann FL, Skjåk-Bræk G. 2019. Biosynthesis and function of long guluronic acid-blocks in alginate produced by Azotobacter vinelandii. Biomacromolecules 20:1613–1622. doi:10.1021/acs.biomac.8b01796 30844259

[26] Gaardløs M, Samsonov SA, Sletmoen M, Hjørnevik M, Sætrom GI, Tøndervik A, Aachmann FL. 2021. Insights into the roles of charged residues in substrate binding and mode of action of mannuronan C-5 epimerase AlgE4. Glycobiology 31:1616–1635. doi:10.1093/glycob/cwab025 33822050

[27] Holtan S, Bruheim P, Skjåk-Braek G. 2006. Mode of action and subsite studies of the guluronan block-forming mannuronan C-5 epimerases AlgE1 and AlgE6. Biochem J 395:319–329. doi:10.1042/BJ20051804 16390328 PMC1422759

[28] Aarstad OA, Strand BL, Klepp-Andersen LM, Skjåk-Bræk G. 2013. Analysis of G-block distributions and their impact on gel properties of in vitro epimerized mannuronan. Biomacromolecules 14:3409–3416. doi:10.1021/bm400658k 23937556

[29] Tøndervik A, Klinkenberg G, Aachmann FL, Svanem BIG, Ertesvåg H, Ellingsen TE, Valla S, Skjåk-Bræk G, Sletta H. 2013. Mannuronan C-5 epimerases suited for tailoring of specific alginate structures obtained by high-throughput screening of an epimerase mutant library. Biomacromolecules 14:2657–2666. doi:10.1021/bm4005194 23808543

[30] Buchinger E, Knudsen DH, Behrens MA, Pedersen JS, Aarstad OA, Tøndervik A, Valla S, Skjåk-Bræk G, Wimmer R, Aachmann FL. 2014. Structural and functional characterization of the R-modules in alginate C-5 epimerases AlgE4 and AlgE6 from Azotobacter vinelandii. J Biol Chem 289:31382–31396. doi:10.1074/jbc.M114.567008 25266718 PMC4223338

[31] Aachmann FL, Svanem BIG, Güntert P, Petersen SB, Valla S, Wimmer R. 2006. NMR structure of the R-module: a parallel beta-roll subunit from an Azotobacter vinelandii mannuronan C-5 epimerase. J Biol Chem 281:7350–7356. doi:10.1074/jbc.M510069200 16407237

[32] Nøkling-Eide K, Aachmann FL, Tøndervik A, Arlov Ø, Sletta H. 2024. In-process epimerisation of alginates from Saccharina latissima, Alaria esculenta and Laminaria hyperborea. Carbohydr Polym 325:121557. doi:10.1016/j.carbpol.2023.121557 38008481

[33] Xu C, Tong S, Sun L, Gu X. 2023. Cellulase immobilization to enhance enzymatic hydrolysis of lignocellulosic biomass: an all-inclusive review. Carbohydr Polym 321:121319. doi:10.1016/j.carbpol.2023.121319 37739542

[34] Lv K, Yu Z, Pedroso MM, Wu B, Gao Z, He B, Schenk G. 2021. Metal affinity immobilization of the processive endoglucanase EG5C-1 from Bacillus subtilis on a recyclable pH-responsive polymer. ACS Sustainable Chem Eng 9:7948–7959. doi:10.1021/acssuschemeng.1c02215

[35] Coscolín C, Beloqui A, Martínez-Martínez M, Bargiela R, Santiago G, Blanco RM, Delaittre G, Márquez-Álvarez C, Ferrer M. 2018. Controlled manipulation of enzyme specificity through immobilization-induced flexibility constraints. Appl Catal A 565:59–67. doi:10.1016/j.apcata.2018.08.003

[36] Secundo F. 2013. Conformational changes of enzymes upon immobilisation. Chem Soc Rev 42:6250–6261. doi:10.1039/c3cs35495d 23482973

[37] Hassan ME, Awad GEA, MohyEldin MS, Haroun BM, El-Diwany AI, Elnashar MM. 2023. Carbohydrate microcapsules tailored and grafted for covalent immobilization of glucose isomerase for pharmaceutical and food industries. Biotechnol Lett 45:175–189. doi:10.1007/s10529-022-03323-1 36482052

[38] Makrydaki E, Donini R, Krueger A, Royle K, Moya Ramirez I, Kuntz DA, Rose DR, Haslam SM, Polizzi KM, Kontoravdi C. 2024. Immobilized enzyme cascade for targeted glycosylation. Nat Chem Biol 20:732–741. doi:10.1038/s41589-023-01539-4 38321209 PMC11142912

[39] Higgins D, Dworkin J. 2012. Recent progress in Bacillus subtilis sporulation. FEMS Microbiol Rev 36:131–148. doi:10.1111/j.1574-6976.2011.00310.x 22091839 PMC3237856

[40] Shuster B, Khemmani M, Abe K, Huang X, Nakaya Y, Maryn N, Buttar S, Gonzalez AN, Driks A, Sato T, Eichenberger P. 2019. Contributions of crust proteins to spore surface properties in Bacillus subtilis. Mol Microbiol 111:825–843. doi:10.1111/mmi.14194 30582883 PMC6417949

[41] Tan IS, Ramamurthi KS. 2014. Spore formation in Bacillus subtilis. Environ Microbiol Rep 6:212–225. doi:10.1111/1758-2229.12130 24983526 PMC4078662

[42] Thorne CB. 1962. Transduction in Bacillus subtilis. J Bacteriol 83:106–111. doi:10.1128/jb.83.1.106-111.1962 13921030 PMC314795

[43] Leggett MJ, McDonnell G, Denyer SP, Setlow P, Maillard JY. 2012. Bacterial spore structures and their protective role in biocide resistance. J Appl Microbiol 113:485–498. doi:10.1111/j.1365-2672.2012.05336.x 22574673

[44] Isticato R, Cangiano G, Tran HT, Ciabattini A, Medaglini D, Oggioni MR, De Felice M, Pozzi G, Ricca E. 2001. Surface display of recombinant proteins on Bacillus subtilis spores. J Bacteriol 183:6294–6301. doi:10.1128/JB.183.21.6294-6301.2001 11591673 PMC100119

[45] Zhang X, Al-Dossary A, Hussain M, Setlow P, Li J. 2020. Applications of Bacillus subtilis spores in biotechnology and advanced materials. Appl Environ Microbiol 86:e01096-20. doi:10.1128/AEM.01096-20 32631858 PMC7440806

[46] Karava M, Gockel P, Kabisch J. 2021. Bacillus subtilis spore surface display of photodecarboxylase for the transformation of lipids to hydrocarbons. Sustainable Energy Fuels 5:1727–1733. doi:10.1039/D0SE01404D

[47] Polayes DA, Parks TD, Johnston SA, Dougherty WG. 1998. Application of TEV protease in protein production, p 169–183. In Reischl U (ed), Molecular diagnosis of infectious diseases. Humana Press, Totowa, NJ. 10.1385/0-89603-485-2:169 21390844

[48] Malhotra A. 2009. Chapter 16 tagging for protein expression, p 239–258. In Burgess RR, Deutscher MP (ed), Methods in enzymology. Academic Press. 10.1016/S0076-6879(09)63016-0 19892176

[49] Carrington JC, Dougherty WG. 1987. Processing of the tobacco etch virus 49K protease requires autoproteolysis. Virology (Auckl) 160:355–362. doi:10.1016/0042-6822(87)90006-7 18644573

[50] Zander M, Schmid J, Kabisch J. 2024. Implementation of spore display in Paenibacillus polymyxa with different hydrolytic enzymes. Microorganisms 12:1438. doi:10.3390/microorganisms12071438 39065206 PMC11278568

[51] Zhang Y, Werling U, Edelmann W. 2012. SLiCE: a novel bacterial cell extract-based DNA cloning method. Nucleic Acids Res 40:e55. doi:10.1093/nar/gkr1288 22241772 PMC3333860

[52] Karava M. 2021. Development of a platform for immobilization of proteins based on Bacillus subtilis spores

[53] Greenleaf AL, Losick R. 1973. Appearance of a ribonucleic acid polymerase-binding protein in asporogenous mutants of Bacillus subtilis. J Bacteriol 116:290–294. doi:10.1128/jb.116.1.290-294.1973 4200841 PMC246421

[54] Leighton TJ, Doi RH. 1971. The stability of messenger ribonucleic acid during sporulation in Bacillus subtilis. J Biol Chem 246:3189–3195. doi:10.1016/S0021-9258(18)62213-6 4995746

[55] Gimmestad M, Sletta H, Ertesvåg H, Bakkevig K, Jain S, Suh S, Skjåk-Braek G, Ellingsen TE, Ohman DE, Valla S. 2003. The Pseudomonas fluorescens AlgG protein, but not its mannuronan C-5-epimerase activity, is needed for alginate polymer formation. J Bacteriol 185:3515–3523. doi:10.1128/JB.185.12.3515-3523.2003 12775688 PMC156231

[56] Ertesvåg H, Skjåk-Bræk G. 1999. Modification of alginate using mannuronan C-5-epimerases, p 71–78. In Bucke C (ed), Carbohydrate biotechnology protocols. Humana Press, Totowa, NJ.

[57] Wishart DS, Bigam CG, Holm A, Hodges RS, Sykes BD. 1995. 1H, 13C and 15N random coil NMR chemical shifts of the common amino acids. I. Investigations of nearest-neighbor effects. J Biomol NMR 5:67–81. doi:10.1007/BF00227471 7881273

[58] Cui Z, Kawada M, Hui Y, Sim S. 2024. Programming aliphatic polyester degradation by engineered bacterial spores. bioRxiv:2024.07.16.603759. doi:10.1101/2024.07.16.603759 PMC11927956 39989420

[59] Hui Y, Cui Z, Sim S. 2022. Stress-tolerant, recyclable, and renewable biocatalyst platform enabled by engineered bacterial spores. ACS Synth Biol 11:2857–2868. doi:10.1021/acssynbio.2c00256 35878063

[60] Wang Q, Liu S, Yang G, Chen J, Ji X, Ni Y. 2016. Recycling cellulase towards industrial application of enzyme treatment on hardwood kraft-based dissolving pulp. Bioresour Technol 212:160–163. doi:10.1016/j.biortech.2016.04.048 27099940

[61] Li G, Huang Z, Zhang C, Dong B-J, Guo R-H, Yue H-W, Yan L-T, Xing X-H. 2016. Construction of a linker library with widely controllable flexibility for fusion protein design. Appl Microbiol Biotechnol 100:215–225. doi:10.1007/s00253-015-6985-3 26394862

[62] Ricca E, Baccigalupi L, Isticato R. 2021. Spore-adsorption: mechanism and applications of a non-recombinant display system. Biotechnol Adv 47:107693. doi:10.1016/j.biotechadv.2020.107693 33387640

[63] Piktel E, Pogoda K, Roman M, Niemirowicz K, Tokajuk G, Wróblewska M, Szynaka B, Kwiatek WM, Savage PB, Bucki R. 2017. Sporicidal activity of ceragenin CSA-13 against Bacillus subtilis. Sci Rep 7:44452. doi:10.1038/srep44452 28294162 PMC5353641

[64] Haug A. 1964. Composition and properties of alginates. N.T.H. Trykk.

[65] Rozeboom HJ, Bjerkan TM, Kalk KH, Ertesvåg H, Holtan S, Aachmann FL, Valla S, Dijkstra BW. 2008. Structural and mutational characterization of the catalytic A-module of the mannuronan C-5-epimerase AlgE4 from Azotobacter vinelandii. J Biol Chem 283:23819–23828. doi:10.1074/jbc.M804119200 18574239 PMC3259796

[66] Mao L, Jiang S, Li G, He Y, Chen L, Yao Q, Chen K. 2012. Surface display of human serum albumin on Bacillus subtilis spores for oral administration. Curr Microbiol 64:545–551. doi:10.1007/s00284-012-0109-4 22411215

