# Supplementary material for: Immobilization of alginate C-5 epimerases using Bacillus subtilis spore display
Source: Appl Environ Microbiol. 2025 Apr 3;91(4):e00298-25. doi: 10.1128/aem.00298-25 (PMC12016494; doi:10.1128/aem.00298-25)
Supplement: Supplemental material — Figures S1 to S3; Tables S1 and S2. [file aem.00298-25-s0001.pdf]

Supplementary information

Figures

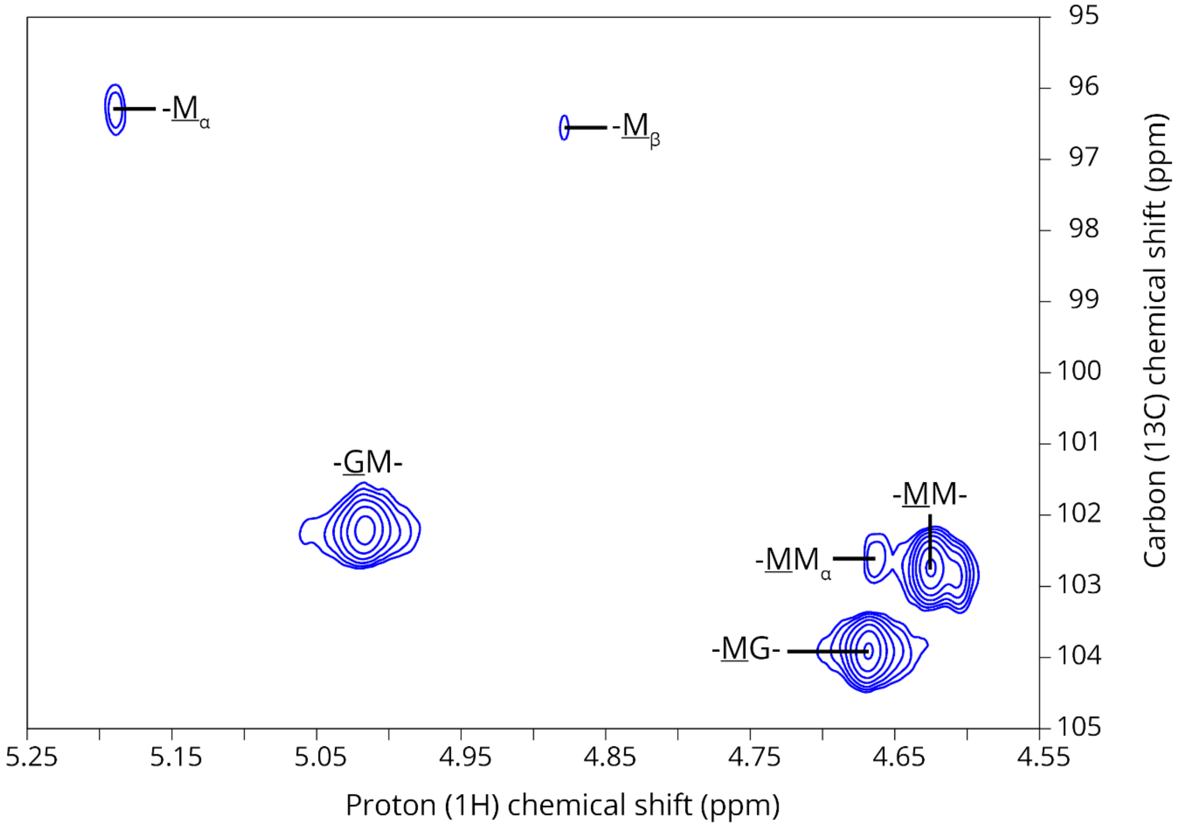

Figure S1 | ^{1}H-^{13}C HSQC after 16 h reaction between AlgE4-C-Rigid and ^{13}C-1 labelled polyM. The figure shows the anomeric region of the spectra, with H/C-1 signals labelled. The assignment was performed according to previous assignments (1-3).

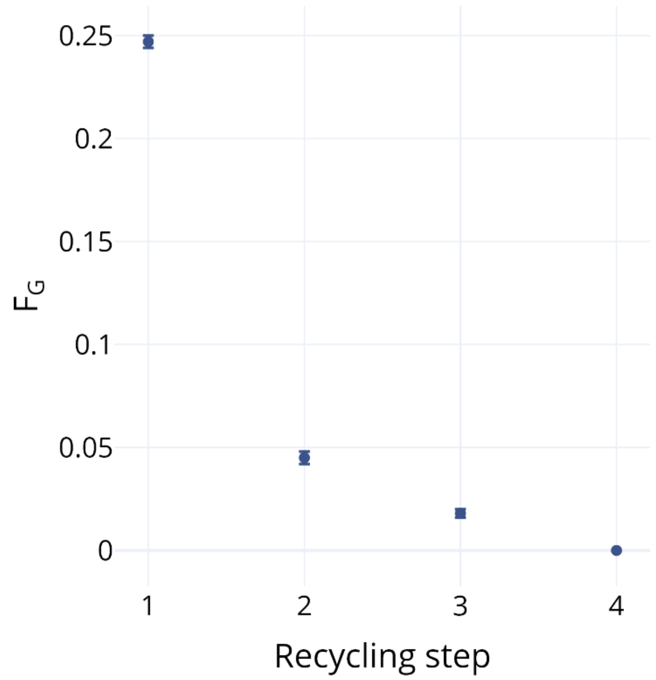

Figure S2 | Reaction between AlgE4-C-Rigid (OD_{600nm} of 3.2) and polyM (6.39 mg/mL), where the same spore pellet is recycled for each reaction. Each reaction was run for 12h. The left axis shows the fraction of G-residues in the product, and the right axis show the activity of the spores as a percentage of the activity of the first reaction. This reaction was run at 50 °C, and the activity drops significantly already after one recycling. After the third recycling of the spores, no activity could be detected. (n=3)

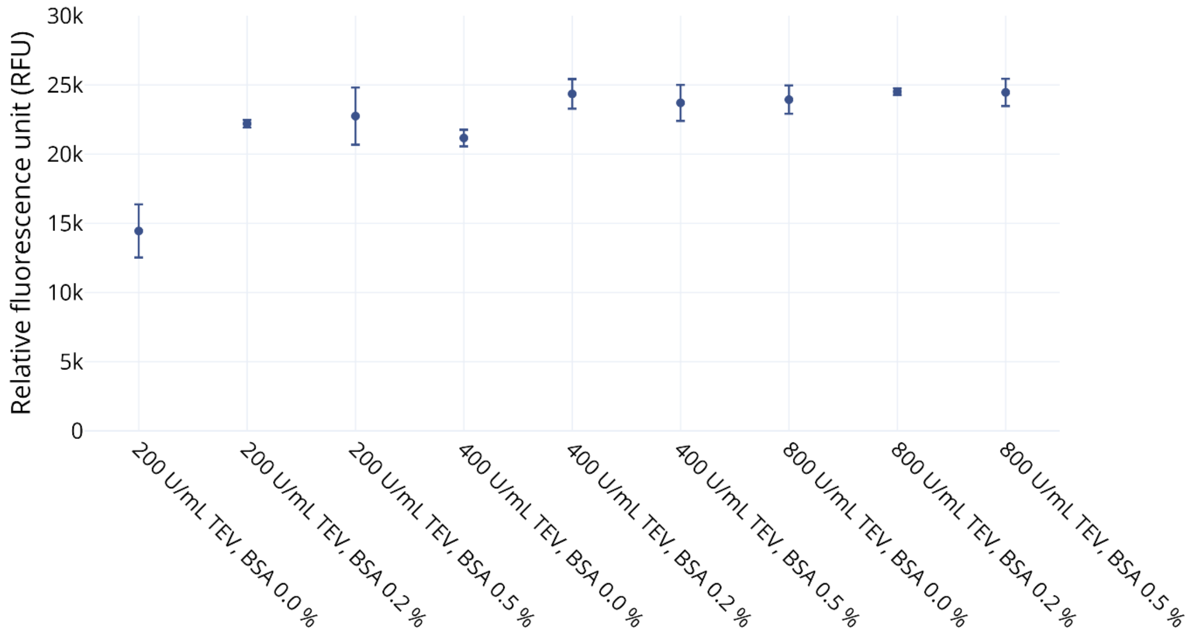

Figure S3 | Measured sfGFP fluorescence in the supernatant after treatment of GFP-N-Flex with TEV. Cleavage was performed for 18 h at 30 °C, spores were separated by centrifugation, and fluorescence of the supernatant was measured. Amount of TEV and BSA was varied to determine the optimal cleavage conditions. (n=3)

Tables

Table S1 | Linker sequencesAmino acid sequences of linkers used in this study to link CotY to the epimerases or GFP.

Abbreviation	Name	Protein sequence
Rigid	semi-rigid linker	EAAAKEAAAKEAAAK
Flex	flexible linker	GGGGSGGGGS

Tabe S2 | Chemicals and suppliers. List of chemicals used in this project along with their suppliers and ordering numbers.

Chemical	Supplier	Ordering number
**General cultivation**		
LB Media (5g/L NaCl)	Sigma-Aldrich	L3022-1kg
**Sporulation**		
Nutrient Broth No.4	Sigma-Aldrich	03856-500g
KCl	Roth	6781.3
$MgSO_4 \cdot 7H_2O$	Roth	P027.1
$Ca(NO_3)_2 \cdot 4H_2O$	Sigma-Aldrich	13477-34-4
$MnCl_2 \cdot 4H_2O$	VWR	25222.233
$FeSO_4 \cdot 7H_2O$		
Glucose	Sigma-Aldrich	G8270-1kg
MOPS	Roth	6979.4
**Spore buffers**		
Lysozyme	Sigma-Aldrich	62971-10G-F
NaCl	Sigma-Aldrich	S9888-1kg-M
Tris-HCl	Roth	9090.3
$CaCl \cdot 2H_2O$	Sigma-Aldrich	12022-1kg
HEPES	Sigma-Aldrich	H4034-100G
DTT	Roth	6908.1
EDTA	VWR	20309.296

References

1. Grasdalen H. 1983. High-field, ^{1}H-N.M.R. Spectroscopy of Alginate: Sequential Structure and Linkage Conformations. Carbohydrate Research 118:255-260.
2. Grasdalen H, Larsen B, Smidsrød O. 1981. ^{13}C-N.M.R. Studies of Monomeric Composition and Sequence in Alginate. Carbohydrate Research 89:179-191.

3. Heyraud A, Gey C, Leonard C, Rochas C, Girond S, Kloareg B. 1996. NMR Spectroscopy Analysis of Oligoguluronates and Oligomannuronates Prepared by Acid or Enzymatic Hydrolysis of Homopolymeric Blocks of Alginic Acid. Application to the Determination of the Substrate Specificity of *Haliotis tuberculata* Alginate Lyase. Carbohydrate Research 289:11-23.
